# Long-term activation of TLR3 by Poly(I:C) induces inflammation and impairs lung function in mice

**DOI:** 10.1186/1465-9921-10-43

**Published:** 2009-06-01

**Authors:** Nicole C Stowell, Jonathan Seideman, Holly A Raymond, Karen A Smalley, Roberta J Lamb, Devon D Egenolf, Peter J Bugelski, Lynne A Murray, Paul A Marsters, Rachel A Bunting, Richard A Flavell, Lena Alexopoulou, Lani R San Mateo, Don E Griswold, Robert T Sarisky, M Lamine Mbow, Anuk M Das

**Affiliations:** 1 Discovery Research, Centocor Research & Development, Inc, Radnor, Pennsylvania, USA; 2 Department of Immunobiology, Yale University School of Medicine and Howard Hughes Medical Institute, New Haven, Connecticut, USA; 3 Centre d'Immunologie de Marseille-Luminy, CNRS-INSERM-Universite de la Mediterranee, Campus de Luminy, Case 906, Marseille Cedex 13288, France; 4 Genomics Institute of the Novartis Research Foundation, San Diego, California, USA

## Abstract

**Background:**

The immune mechanisms associated with infection-induced disease exacerbations in asthma and COPD are not fully understood. Toll-like receptor (TLR) 3 has an important role in recognition of double-stranded viral RNA, which leads to the production of various inflammatory mediators. Thus, an understanding of TLR3 activation should provide insight into the mechanisms underlying virus-induced exacerbations of pulmonary diseases.

**Methods:**

TLR3 knock-out (KO) mice and C57B6 (WT) mice were intranasally administered repeated doses of the synthetic double stranded RNA analog poly(I:C).

**Results:**

There was a significant increase in total cells, especially neutrophils, in BALF samples from poly(I:C)-treated mice. In addition, IL-6, CXCL10, JE, KC, mGCSF, CCL3, CCL5, and TNFα were up regulated. Histological analyses of the lungs revealed a cellular infiltrate in the interstitium and epithelial cell hypertrophy in small bronchioles. Associated with the pro-inflammatory effects of poly(I:C), the mice exhibited significant impairment of lung function both at baseline and in response to methacholine challenge as measured by whole body plethysmography and an invasive measure of airway resistance. Importantly, TLR3 KO mice were protected from poly(I:C)-induced changes in lung function at baseline, which correlated with milder inflammation in the lung, and significantly reduced epithelial cell hypertrophy.

**Conclusion:**

These findings demonstrate that TLR3 activation by poly(I:C) modulates the local inflammatory response in the lung and suggest a critical role of TLR3 activation in driving lung function impairment. Thus, TLR3 activation may be one mechanism through which viral infections contribute toward exacerbation of respiratory disease.

## Background

The activation of Toll-Like Receptors (TLRs), a family of innate immune receptors, is believed to be an important step in the initiation of the inflammatory response raised against numerous pathogens. TLR3 is a mammalian pattern recognition receptor that recognizes double-stranded (ds) RNA as well as the synthetic ds RNA analog poly-riboinosinic-ribocytidylic acid (poly(I:C)) [1]. Activation of TLR3 by poly(I:C) or by endogenous mRNA ligands, such as those released from necrotic cells [2], induces secretion of pro-inflammatory cytokines and chemokines, a finding that suggests that TLR3 agonists modulate disease outcome during infection-associated inflammation [3]. Thus, long-term activation of TLR3 *in vivo *is thought to occur in the context of viral infection [4] or necrosis associated with inflammation [2].

*In vitro *studies have demonstrated that stimulation of lung epithelial cells with poly(I:C) elicited the secretion of multiple cytokines, chemokines, the induction of transcription factors and increased expression of TLRs [3]. It has also been demonstrated that poly(I:C) enhanced bradykinin- and [des-Arg^9^]-bradykinin-induced contractions of tracheal explants *in vitro*, an effect mediated by C-jun-amino-terminal kinase (JNK) and nuclear factor kappa B (NF-kB) signaling pathways [5]. Taken together, these data suggest that TLR3 activation may have a physiological consequence in the lung. Further, these data demonstrate that ligation of TLR3 initiates cascades of phosphorylation and transcriptional activation events that result in the production of numerous inflammatory cytokines that are thought to contribute to innate immunity [5]. Overall, these data suggest that sustained TLR3 activation can be a critical component in the modulation of infection-associated inflammatory diseases.

Exacerbations in respiratory diseases such as asthma and chronic obstructive pulmonary disease (COPD) are characterized by the worsening of symptoms and a decline in lung function. Viral infections are associated with respiratory disease exacerbations [6] and may be associated with progression of disease. Secretion of pro-inflammatory cytokines in the lungs following viral infection represents a crucial step in promoting the inflammatory response in various lung diseases [7,8]. A better understanding of the effects of TLR3 activation may provide insight into the mechanisms underlying virally-induced respiratory disease exacerbations.

In the current study we examined the effects of TLR3 activation *in vivo*. We sought to induce long term activation of TLR3 to mimic the physiologic disease state associated with virally-induced disease exacerbations. Administration of poly(I:C) to the lungs of mice induced a marked impairment of lung function that was accompanied by the production of pro-inflammatory mediators and inflammatory cell recruitment into the airways. TLR3 appears to play a role in the effects of poly(I:C) since TLR3 KO mice were partially protected. Taken together, our data suggest an important role for TLR3 activation in impairment of lung function.

## Methods

### Poly(I:C) induced cytokine secretion in BEAS-2B cells

The SV-40-transformed normal human bronchial epithelial cell line, BEAS-2B (ATCC, VA) was cultured in LHC-9 media without additional supplements. (Biosource, CA). 1 × 10^6 ^cells were seeded in collagen type I-coated T75 flasks (BD, NJ) and split every 2–3 days using 0.25% trypsin/ethylenediaminetetraacetic acid (EDTA) (Gibco, CA). Poly(I:C) (Amersham, NJ) was dissolved in phosphate-buffered saline (10 mM phosphate, 150 mM NaCl, pH 7.4; phosphate buffered saline (PBS)) at a concentration of 2 mg/ml and aliquots were stored at -20°C. For poly(I:C) stimulation, cells were incubated at 37°C with different concentrations of poly(I:C). Supernatants were collected after 24 hours and stored at -20°C or assayed immediately for cytokine secretion using a multi-plex bead assay (Biosource, CA) for detection of interferon-alpha (IFNα), interferon-gamma (IFNγ), interleukin-1-beta (IL-1β), interleukin-10 (IL10), interleukin-12p70 (IL12p70), tumor necrosis factor-alpha (TNFα), Chemokine (C-C motif) ligand 3 (CCL3), interleukin-6 (IL-6), interleukin-8 (IL-8), Chemokine (C-C motif) ligand 2 (CCL2), Chemokine (C-C motif) ligand 5 (CCL5), and Chemokine (C-X-C motif) ligand 3 (CXCL10). Limits of detection for the analytes range from 3 – 20 pg/ml. Sample acquisition and analysis was performed using the Luminex 100S with StarStation software (Applied Cytometry Systems).

### Administration of Poly(I:C) to the lungs of mice

Female C57BL/6 mice wild-type (WT) (12 weeks old) or female TLR3 knock-out (KO) mice (C57BL/6; 12 weeks old, ACE animals, PA) were anesthetized with isoflurane and different doses (10–100 μg) of poly(I:C) in 50 μl sterile PBS, or PBS alone, were administered intranasally (I.N.) Mice received three administrations of poly(I:C) (or PBS) with a 24 hour rest period between each administration. KO mice were fully backcrossed to C57BL/6 background to at least N10.

All animal care was performed according to the Guide for the Care and Use of Laboratory animals and the Institutional Animal Care and Use Committee approved all studies.

### Whole Body Plethysmography

Twenty-four hours following the last poly(I:C) (or PBS) administration, lung function without provocation (baseline) and airway hyperresponsiveness (AHR) to methacholine were measured using whole body plethysmography (BUXCO system). The mice were placed into the whole body plethysmograph chamber and allowed to acclimate for at least 5 minutes. Following baseline readings, mice were exposed to increasing doses of nebulized methacholine (Sigma, MO). The nebulized methacholine was administered for 2 minutes, followed by a 5-minute data collection period, followed by a 10-minute rest period before subsequent increasing-dose methacholine challenges. The increased airflow resistance was measured as Enhanced Pause (Penh) and is represented as the average penh value over the 5-minute recording period.

### Invasive measures of lung function

Twenty-four hours following the last poly(I:C) (or PBS) administration, lung function and increased lung resistance in response to methacholine were measured using invasive measures of lung function (BUXCO system). Mice were anesthetized with 50 mg/kg sodium pentobarbital (Nembutal, Abbot Labs, IL). The trachea was cannulated with a 19 gauge cannula and the mouse was connected to a mechanical ventilator, with breath frequency of 120 and stroke volume of 0.3 mL. The mouse was connected to the plethysmograph for lung function measurements. After establishing a stable baseline of lung resistance, methacholine was administered I.V. through the tail vein (240 μg/kg). The peak resistance measured over 3 minutes was recorded.

### Measurement of lung inflammation

Following lung function measurements, mice were sacrificed by CO_2 _asphyxiation and the lungs were cannulated. Bronchoalveolar lavages (BAL) were performed by injecting 1 mL of PBS into the lungs and retrieving the effluent. The lung tissues were removed and frozen. The BALs were centrifuged (1200 rpm, 10 minutes) and the cell-free supernatants were collected and stored at -80°C until analysis. The cell pellet was resuspended in 200 μl PBS for total and differential cell counts using a hemacytometer (on Wright's – Giemsa-stained cytospin preparations).

### Measurement of proteins in bronchoalveolar lavage samples

The cell-free supernatants were collected and stored at -80°C until used for analyses. The multiplex assay was performed following the manufacturer's protocol and the LINCOplex Multiplex Immunoassay Kit (LINCO Research, St. Charles, MO). Analytes included in the analysis were MIP1α, Granulocyte Macrophage Colony Stimulating Factor (GMCSF), JE, KC, RANTES, IFNγ, IL-1α, IL-1β, Granulocyte Colony Stimulating Factor (GCSF), CXCL10, IL-2, IL-4, IL-5, IL-6, IL-7, IL-9, IL-10, IL-12(p70), IL-13, IL-15, IL-17 and TNFα. Limits of detection for the analytes range from 3 – 20 pg/ml.

### Measurement of lung mRNA expression

Following collection of BAL samples, the right lobes of the lung were removed and placed in Trizol total RNA isolation reagent (Life Technologies, Gaithersburg, MD). RNA was isolated using manufacturer's instructions of the Qiagen Rneasy Mini kit (Qiagen, Valencia, CA). Total RNA (2 μg) from pooled groups was then reverse transcribed using the OmniScript RT kit (Qiagen, Valencia, CA) according to the manufacturer's protocol. One hundred nanograms of cDNA was then amplified using both the TaqMan^® ^Low Density Immune Profiling Array cards (Applied Biosystems, Foster City, CA), or microfluidic cards, and custom Low Density Array cards. Primer-probes with genes of interest were plated in a 384 well format following the manufacturer's protocol for Real-Time PCR. Data are normalized to 18s rRNA and represent fold change over PBS treated mice.

### Histological Analysis

Following BAL collection, the left lobes were inflated with 10% neutral buffered formalin under constant pressure then immersed in additional fixative, the right lobes were clamped with hemostats and ligated. Tissue was processed by routine methods, oriented so as to provide coronal sections and 5 micron mid-coronal sections cut and stained with hematoxylin and eosin.

### Morphometric analysis

A Nikon Eclipse E800 (Nikon Corporation, Tokyo, Japan) microscope was equipped with an Evolution™ MP 5.0 RTV color camera (Media Cybernetics, Inc. Silver Spring, MD). Images were captured and analyzed using Image-Pro Plus software version 5.1 (Media Cybernetics, Inc. Silver Spring, MD). GraphPad Prism version 4.03 (GraphPad Software, Inc. San Diego, CA) was used to interpret, analyze and graph the raw data. SigmaStat Statistical Software version 2.03 (SPSS, Inc. Chicago, IL) was used to perform statistical analysis on the collected data. Using the Auto-Pro tool within the Image-Pro Plus software, custom written macros were used to perform the analysis. Six TLR3 KO mice treated with poly(I:C), six WT mice treated with poly(I:C), four TLR3 KO mice treated with PBS and six WT mice treated with PBS were imaged and analyzed. No imaging or analysis was performed on areas of the lung that were torn, damaged, or folded.

### Tissue Density

From each lung, five fields were randomly selected and imaged using a 20× objective lens. The total area of the tissue was measured and the ratio of total area of tissue to total area of field calculated.

### Tissue Cellularity

From each lung, five fields were randomly selected and imaged using a 20× objective lens. The total area of the nuclei was measured and the ratio of total area of nuclei to total area of field calculated.

### Airway Cellularity

From each lung, five airways were chosen and imaged using a 40× objective lens. A line of 100 μm in length was superimposed on the airway at a random location. The number of nuclei within the fixed distance were counted and recorded.

### Airway Mucosal Height

From each lung, five airways were chosen and imaged using a 40× objective lens. The image was segmented so as to include only the airway mucosa and the average thickness of the airway mucosa was measured using the curve thickness algorithm built into ImagePro. This algorithm parses the mucosa into 30,000 arc segments, measures the thickness of the mucosa at each arc segment and calculated the average thickness for the mucosa.

### Statistical analysis

Specific statistical methods are described in the figure legends. Graphs and summary statistics were also used to assess the results. All statistical tests were 2-sided. Except for where noted, all p-values presented are unadjusted for multiple comparisons.

## Results

### Poly(I:C) induces a marked inflammatory response in the lungs of mice

**Figure 1:**
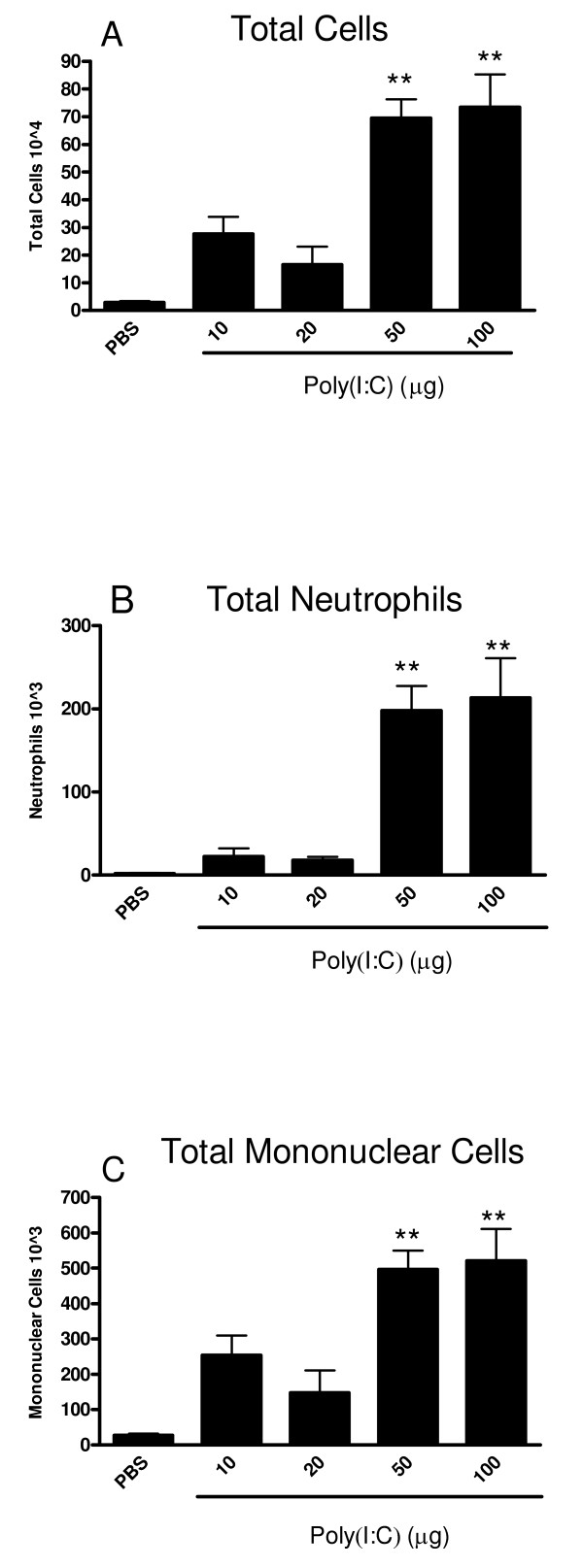
**Poly(I:C) induces a dose dependent influx of inflammatory cells into the airways of mice**. Mice were administered PBS or, 10, 20, 50 or 100 μg poly(I:C) (I.N.) every 24 h for three days. 24 hours after the last administration, mice were euthanized and BALs were performed. The total number of cells (1A), neutrophils (1B) and mononuclear cells (1C) were measured in the BAL. Data are the mean ± SEM of 6–15 mice from two separate experiments. The Kruskal-Wallace test was used to compare the treatment groups. When this test showed a difference among the treatment groups, selected pairs of treatments were compared using Dunn's multiple comparison test. ** p < 0.001 when compared to PBS-treated mice.

Intranasal administration of three once-daily doses of poly(I:C) resulted in a dose-dependent inflammatory cell influx into the lung. There was a significant increase in total cells in the BAL samples at 50 and 100 μg poly(I:C) compared to PBS treated mice (Figure 1A). This increase in total cellularity in the BAL samples was partially due to a significant influx of neutrophils (Figure 1B) and mononuclear cells (Figure 1C). Due to the robust response at 50 and 100 μg, these doses of poly(I:C) were used in our subsequent studies.

In an effort to understand the responses to poly(I:C) treatment in the lung at a molecular level, Taqman real-time PCR analyses of the lung tissues was performed. Multiple administrations of poly(I:C) elicited up regulation of a number of pro-inflammatory genes, TLRs and their associated intracellular signaling molecules (Table 1). TLR genes that were up regulated at the mRNA level as a result of TLR3 stimulation included TLR2, TLR3, TLR7, and TLR9 with approximately 7, 5, 11, and 56 fold increases respectively. In addition there was dramatic increase in CXCL10, TNFα, CCL2, CCL3, and CCL7 gene expression as well as interferon regulatory factor 7 (IRF7), interferon-stimulated transcription factor 3 (ISGF3G), 2'-5'-oligoadenylate synthetase 2 (OAS2), and protein kinase-R (PKR.)

**Table 1:** Poly(I: C) induces up regulation of gene expression of cytokines, chemokines, signaling molecules and TLRs in the lungs of mice.

**Cytokines/Chemokines**	**Fold Increase**
CXCL10	357.38
TNFα	78.45
CCL2	76.62
CCL3	30.49
CCL7	48.38
**TLRs**	
TLR9	55.78
TLR7	10.86
TLR3	5.41
TLR2	6.96
**Transcription Factors**	
IRF7	22.92
ISGF3G	4.45
Enzymes	
OAS2	10.76
PKR	9.32

Mice were administered PBS or 100 μg poly(I:C) I.N. every 24 h for three days. 24 h following the last poly(I:C) administration, lungs were lavaged, excised and frozen. RNA was isolated from the tissue and real-Time PCR was then performed. Data are expressed as fold change in mRNA expression over PBS-treated animals and represent pooled cDNA from 6 – 8 mice.

Poly(I:C) administration also induced elevated protein levels of cytokines, chemokines, and growth factors in the lavage including significant increases of IFNγ, IL-1α, IL-6, TNFα, CXCL10, JE, KC, MIP-1α, RANTES, GCSF and GMCSF (Table 2). There were no changes in IL-1β, IL-2, IL-4, IL-5, IL-7, IL-9, IL-10, IL-12(p70), IL-13, IL-15, or IL-17 (data not shown) among the groups. These data demonstrate that poly(I:C) administered I.N. elicits a cascade of events resulting in the expression and secretion of multiple pro-inflammatory cytokines, and chemokines as well as the up regulation of TLR gene expression.

**Table 2:** Poly(I: C) induces the secretion of cytokines, chemokines, and growth factors into the airways.

	Treatment	
Protein (pg/ml)	PBS	100 μg Poly(I:C)
IFNγ	11.0 +/- 1.6	52.2 +/- 11.2 **
IL-1α	16.5 +/- 1.2	21.8 +/- 1.4 *
IL-6	8.8 +/- 1.5	879.0 +/- 171.2 **
CXCL10	30.3 +/- 5.9	411.3 +/- 34.9 **
JE	11.7 +/- 1.2	798.7 +/- 182.6 **
KC	6.2 +/- 1.3	55.4 +/- 6.5 **
GCSF	5.2 +/- 0.7	60.6 +/- 6.8 **
MIP1α	37.7 +/- 6.3	441.1 +/- 61.6 **
RANTES	0.5 +/- 0.04	155.8 +/- 41.6 **
TNFα	2.3 +/- 0.33	81.2 +/- 13.7 **
GMCSF	19.1 +/- 2.1	33.5 +/- 4.5 *

Mice were administered PBS or 100 μg polyI:C (I.N.) every 24 h for three days. 24 h following the last polyI:C administration, BALs were performed. Analyte levels in BAL were determined. Data are expressed as mean pg/ml ± SEM from 6 – 8 mice. Statistical significance was determined using the Mann-Whitney test. * p < 0.05, **p < 0.01 when compared to PBS-treated mice. There was no measureable change in the following cytokines (data not shown): IL-1β, IL-2, IL-4, IL-5, IL-7, IL-10, IL-12(p70), IL-9, IL-13, IL-15, or IL-17.

**Figure 2:**
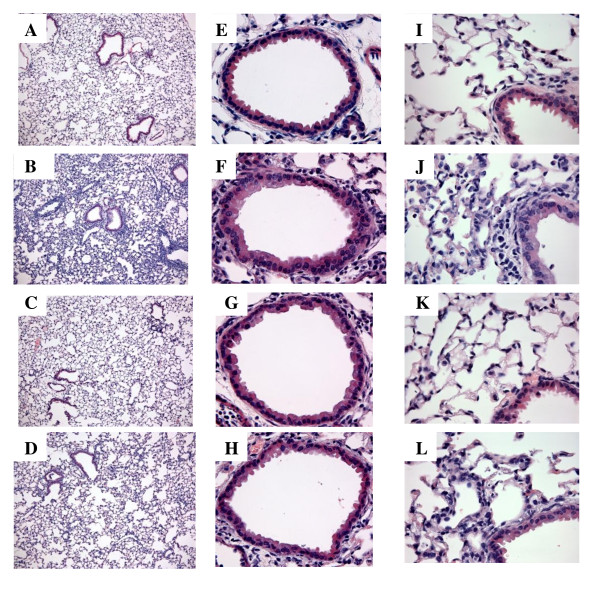
**TLR3 KO mice are partially protected from poly(I:C)-induced inflammation in lung interstitium**. Representative H&E-stained lung sections from WT- PBS treated (A,E, I)WT poly(I:C)-treated (B, F, J), TLR3 KO PBS treated mice (C ,G, K) and TLR3 KO poly(I:C)-treated (D, H, L). Figures A-L are representative images from each group. Figure A-D are at 10×, Figures E-H are at 40 × and Figures I-L are at 60 ×.

Histological analyses of the lungs were performed to better understand the pathology induced by poly(I:C) administration. Representative micrographs from H&E stained lung sections are shown (Figure 2). The histology of the control lungs was unremarkable in that the lungs exhibited normal pulmonary architecture and resident cells. The most remarkable changes induced by poly(I:C) were a marked perivascular and a moderate peribronchiolar interstitial inflammatory infiltrate. There were also signs of pulmonary edema as evidenced by a widening of the interstitial space surrounding the airways and vasculature in the poly(I:C) treated mice. The alveolar septa were thickened and contained numerous inflammatory cells, consistent with an interstitial pneumonitis. Few inflammatory cells were observed in the alveolar spaces, but as the bronchoalveolar fluids were collected, most of the cells in the alveoli were probably lost from analysis. The other remarkable changes observed were thickening of the bronchiolar epithelium consistent with hypertrophy. The hypertrophy was accompanied by an increase in the granularity of the cytoplasm of the bronchiolar epithelium, however, there was no evidence for increased mucus production by PAS staining. There was no notable increase in goblet cells.

The results of the morphometric analysis are shown in Table 3. Reflecting the increase in interstitial penumonitis there was a 1.7 fold increase in tissue density and a 2 fold increase in overall tissue cellularity. In the small airways, there was a 1.7 fold increase in the mucosal height, reflecting the mucosal hypertrophy and no change in cellularity (data not shown).

**Table 3:** Morphometric analysis of lungs from WT PBS control and poly(I:C)-treated, and TLR-3 KO PBS control and poly(I:C)-treated mice.

Group	Tissue Density %	Tissue Cellularity %	Airway Mucosal Height μm
WT PBS	32 ± 2	8 ± 1	15 ± 1
WT Poly(I:C)	50 ± 5*	16 ± 2*	26 ± 4*
Fold Increase (Compared to WT PBS)	1.7	2	1.7
KO PBS	36 ± 7	9 ± 1	18 ± 3
KO Poly(I:C)	49 ± 6*	14 ± 3*	19 ± 2**
Fold Increase (Compared to KO PBS)	1.4	1.5	NC

NC = No change, * Different from respective PBS control. ** Different from poly(I:C)-treated WT. p < 0.01 using T-test to compare groups.

### Poly(I:C) activates BEAS2B epithelial cells

**Figure 3:**
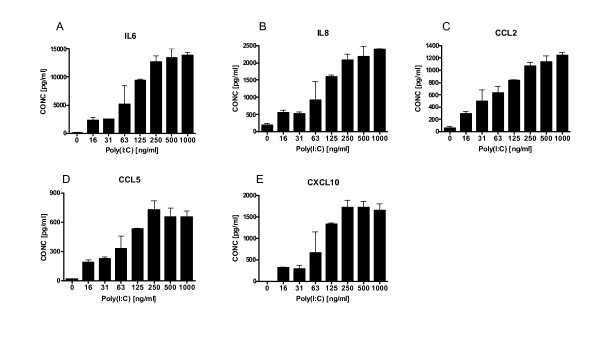
**Poly(I:C) induces cytokine secretion from BEAS-2B cells**. BEAS-2B cells were incubated for 24 hours at 37°C with serial dilutions of polyI:C. Supernatants were collected after 24 hours and assayed for cytokine levels of IL-6 (A), IL-8 (B), CCL2 (C), CCL5 (D), and CXCL10 (E). Data is representative of 2 different experiments.

The morphometric data identified the induction of mucosal hypertrophy in WT mice following poly(I:C) challenge. To further elucidate the effects of poly(I:C) on epithelial cells, the response of the normal human lung epithelial cell line, BEAS-2B, to poly(I:C) was investigated. Similar to the mouse in vivo data, where analysis was performed 24 hours post final poly(I:C) challenge, BEAS-2B cells responded to a range of poly(I:C) concentrations (16 to 1000 ng/ml) in a dose-dependent manner by secreting a number of cytokines observed in the mouse lungs including IL-6, IL-8, CCL2, CCL5, and CXCL10 (Fig. 3), consistent with previous findings [9-11]. There was no change in response to poly(I:C) in the other analytes included in the multiplex (data not shown), nor was there any obvious change in morphometric parameters of the stimulated cells.

### TLR3 stimulation leads to impairment of pulmonary function

**Figure 4:**
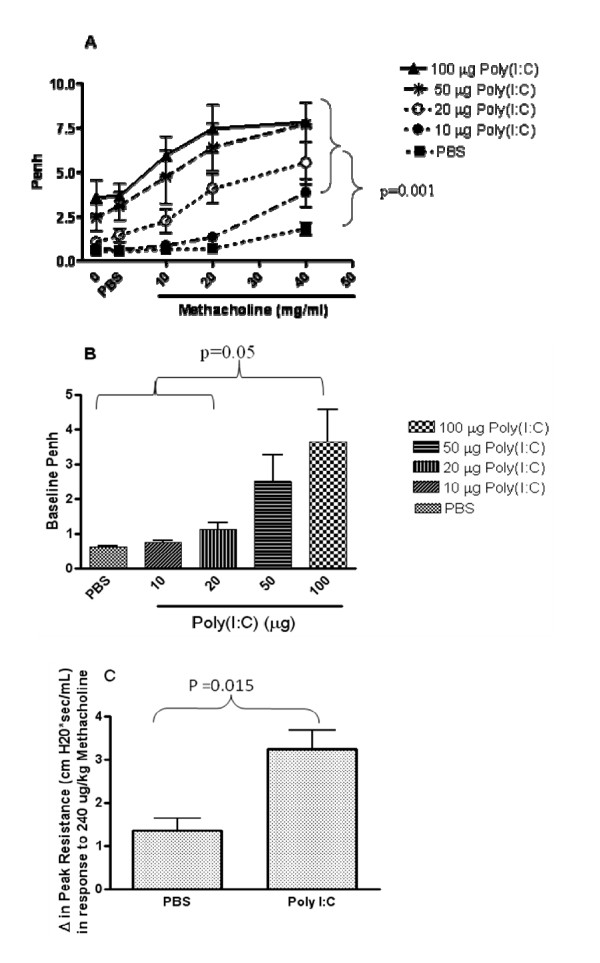
**Poly(I:C) induces impairment of lung function and AHR**. Mice were administered PBS or 10, 20, 50 or 100 μg polyI:C (I.N.) every 24 h for three days. 24 h after the last poly(I:C) administration, baseline lung function and AHR to increasing doses of methacholine was measured by whole body plethysmography (A & B). The 100 ug poly I:C group had higher penh levels than the PBS, 10, and 20 ug groups, p < 0.05 (B). Methacholine challenge resulted in a larger increase from baseline in the poly(I:C)-treated groups than in the PBS group, p < 0.001 for each methacholine dose. Invasive measurements of lung function were performed 24 h following three administrations (24 h apart) of 100 μg poly(I:C) (C). Peak airway resistance after i.v. injection of methacholine at 240 ug/kg are shown. Methacholine challenge resulted in a larger increase from baseline in the poly(I:C)-treated group than in the PBS group, p = 0.015. Repeated measures ANOVA was used to assess the Penh values over increasing methacholine doses as well as to compare increases in resistance in response to methacholine from baseline among the groups. Data are the mean ± SEM of 5–7 mice.

In order to investigate the functional consequences of TLR3 ligation, we measured lung function in poly(I:C)-treated mice. Airway hyperresponsiveness to increasing doses of methacholine was measured using whole body plethysmography (WBP) (Figure 4A). Poly(I:C)-challenged mice exhibited greater airway hyperresponsiveness to methacholine. Poly(I:C)-challenged mice also exhibited an increase in baseline penh in the absence of provocation, measured using WBP (Figure 4B). To confirm the effects of poly(I:C) on lung function, invasive lung function measurements were also performed and the results confirmed those obtained using WBP (Fig 4C).

### Poly(I:C)-induced inflammatory cell influx is attenuated in TLR3 KO mice

**Figure 5:**
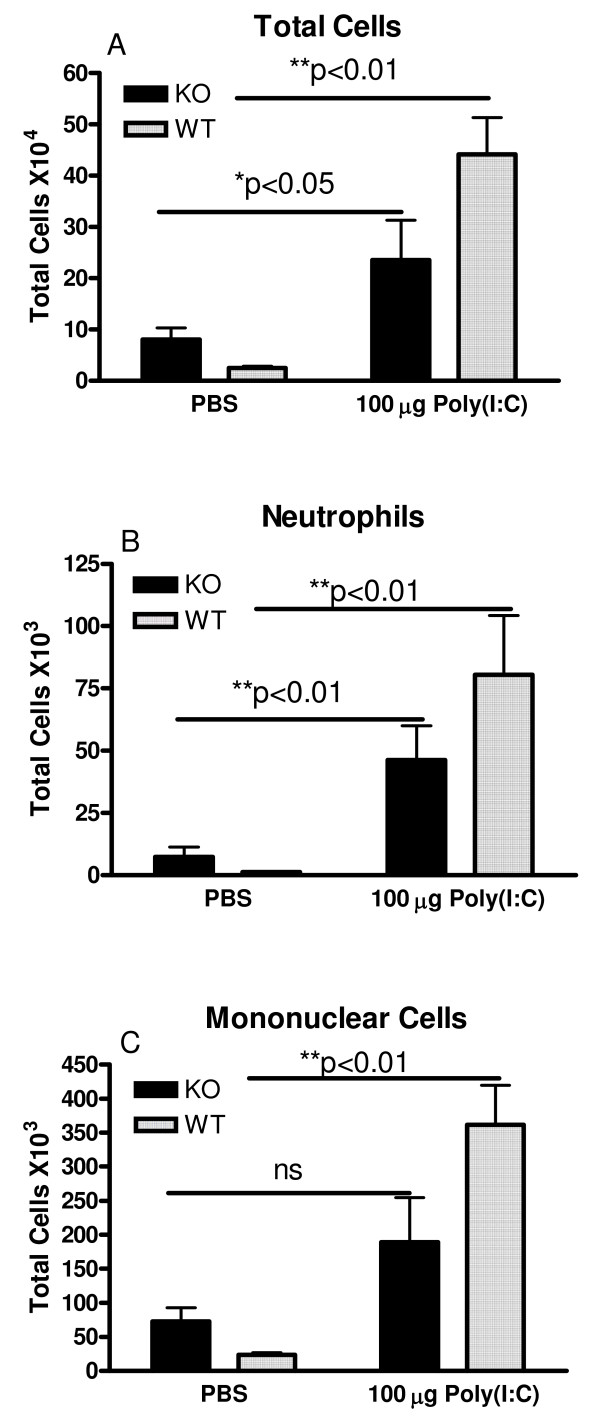
**TLR3 KO mice are partially protected from poly(I:C)-induced inflammatory cell influx in the airways**. Mice were administered PBS or 100 μg poly(I:C) I.N. every 24 h for three days. 24 hours after the last poly(I:C) administration, mice were euthanized and the lungs were lavaged. The total number of cells (5A), neutrophils (5B) and mononuclear cells(5C) were measured in the BAL. Data are the mean ± SEM of 6 mice. Treatment groups (PBS or 100 μg poly(I:C)) and mouse types were compared using 2-way ANOVA, including an interaction term. *p < 0.05, **p < 0.01 compared to PBS-treated mice. When comparing the impact of poly(I:C) treatment on cell populations in the lavage, there was a significantly larger increase in the response of wild type mice than knockout mice, with respect to total cells and mononuclear cells alone, **p < 0.01 in each case. Similar trends were observed in neutrophils alone but failed to reach statistical significance (p = 0.056).

In order to elucidate whether the effects induced by poly(I:C) were mediated through TLR3, we treated TLR3 KO and age-matched WT control mice with three repeated doses of 100 μg poly(I:C) I.N. 24 hours after the third dose, mice were euthanized and bronchoalveolar lavage samples were collected. There was a significant increase in total cells, including both neutrophils and mononuclear cells in the bronchoalveolar lavage samples harvested from WT mice administered 3 doses of 100 μg poly(I:C) compared to PBS treated mice (Figure 5A–C). In contrast, TLR3 KO mice displayed a reduced influx of inflammatory cells compared to WT mice. The increase in total cells, neutrophils, and mononuclear cells in poly(I:C)-treated WT mice was 18, 70, and 15 fold over PBS treated mice respectively. In contrast, poly(I:C)-treated TLR3 KO mice had increases of 3, 6, and 3 fold in total cells, neutrophils, and mononuclear cells over PBS treated TLR3 KO mice.

### TLR3 KO mice are protected from poly(I:C)-induced bronchial epithelial cell hypertrophy

Representative micrographs from H&E stained lung sections from control and poly(I:C)-treated TLR3 KO mice are shown in Figure 2. The histology of the control lungs was largely unremarkable. However, focal eosinophilic mixed inflammatory infiltrates were observed in 2 of 4 TLR3 KO mice examined. The ranges of changes observed in the TLR3 KO mice treated with poly(I:C) was similar to that observed in wild type mice (described above). Perivascular and peribronchiolar interstitial chronic inflammatory infiltrates were present in these mice but were somewhat less extensive. The pulmonary edema and interstitial pneumonitis were modestly attenuated and the bronchiolar epithelial hypertrophy observed in the wild type mice treated with Poly(I:C) was markedly attenuated in the TLR3 KO mice.

The attenuation of the effects of poly(I:C) is corroborated by the morphometric analysis (Table 3). Although there was only a slight change in tissue density in the KO mice compared to WT, the bronchiolar epithelial hypertrophy was decreased substantially.

### TLR3 KO mice are protected from poly(I:C)-induced changes in lung function at baseline

**Figure 6:**
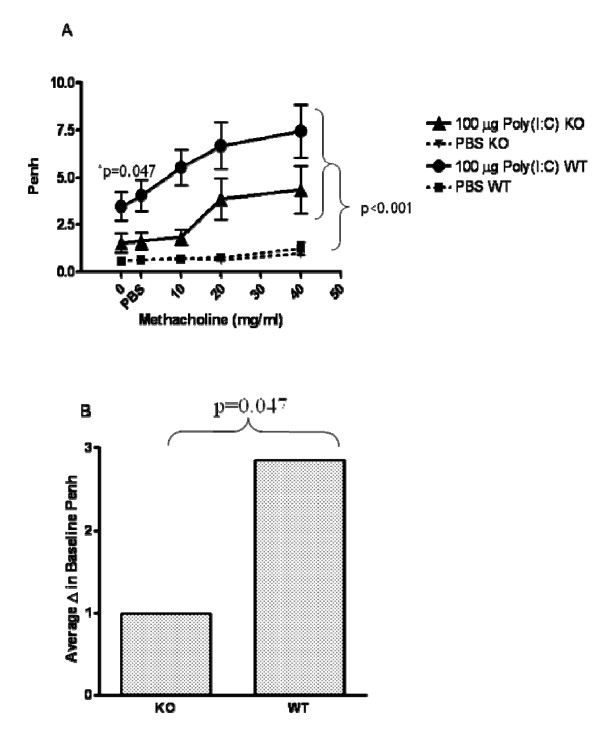
**TLR3 KO mice are partially protected from poly(I:C)-induced impairment of lung function and AHR**. Mice were exposed to three doses of 100 mg poly(I:C) (I.N.; 24 h apart). Baseline lung function and AHR to increasing doses of methacholine was measured by whole body plethysmography 24 hours following the last dose of poly(I:C). Data are the mean ± SEM of 6 mice. Prior to challenge, the groups given poly(I:C) had higher Penh values than those given PBS, p < 0.001. This difference was greater in the WT mice than in the KO mice, p = 0.047. Increasing methacholine challenges lead to higher mean penh values for the Poly I:C treated groups than for the PBS groups, p < 0.001, but there was not a statistically significant difference between the poly I:C-treated KO and WT groups p = 0.115. A repeated measure ANOVA was used to assess the change from pre-challenge penh values over increasing methacholine doses. ANOVA was used to compare the peak resistance levels at baseline among the groups.

In order to investigate whether TLR3 plays a role in poly(I:C)-induced lung function impairment, lung function was measured following poly(I:C) treatment of TLR3 KO mice and WT age-matched controls. As shown in Figure 6B, TLR3 KO mice were protected from poly(I:C)-induced changes at baseline. The increase in penh observed at baseline following poly(I:C) administration was significantly reduced in TLR3 KO mice.

## Discussion

Exacerbations of respiratory diseases such as asthma and COPD are often associated with concomitant respiratory viral infections. Since TLR3 is activated by viral dsRNA, the purpose of the current study was to better understand the functional consequences of TLR3 activation in vivo. Administration of poly(I:C), a synthetic TLR3 ligand, to the lungs of mice induced marked inflammation accompanied by impaired lung function. TLR3 KO mice were partially protected from the effects of poly(I:C) demonstrating the involvement of TLR3. These data provide further support for a role of TLR3 in respiratory diseases and suggest a potential mechanisitic pathway for viral exacerbations.

Upon activation, TLR3 recruits a Toll-IL-1 receptor (TIR) – related adaptor protein inducing interferon (TRIF), which activates both IFN-regulatory factor 3 (IRF3) and NF-kB [12] and [13]. In our model, following poly(I:C) administration to the lungs, there was an up regulation of TLR3, -2, -7, and 9 gene expression and their associated signaling molecules. Previous *in vitro *studies have demonstrated that activation of TLR3 with poly(I:C) induces up regulation of its own expression as well as the expression of other TLRs. For example, poly(I:C) up regulates mRNA for TLR2, 3 and 4 in airway smooth muscle cells [14] and TLR2, 3, 6 and 10 in lung epithelial cells [3]. *In vivo*, the up regulation of TLR mRNA expression may have occurred as a result of expression of TLRs on infiltrating cells or through up regulation on resident lung cells. Indeed, monocytes express all of the known TLRs [15]. In contrast, neutrophils have been shown to express all the TLRs except TLR3 [16]. Within the lung, all of the known TLRs have been found to be expressed by human primary bronchial epithelial [3] and smooth muscle cells [14]. The up regulation of multiple members of the TLR family, as a consequence of activation of one TLR, may indicate the creation of an environment of hyper-responsiveness to pathogen insult whereby, an exacerbation event could be triggered in the event that the lung is exposed to other toll-ligands. In support of this hypothesis, it has been shown that infection of airway epithelial cells with *Hemophilus influenza *induced the secretion of CXCL-8, up regulated TLR3 expression and increased the responsiveness to a secondary challenge of *Rhinovirus*. Interestingly, inhibition of TLR3 with small interfering RNA, inhibited the *Rhinovirus*-induced CXCL-8 production [17]. In addition this same group demonstrated that pretreatment with *Rhinovirus *resulted in delayed bacterial clearance when a secondary infection was induced using nontypeable *Hemophilus influenza*. Sajjan et al. showed that this may be the result of decreases in transepithelial resistance or compromised tight junctions and loss of zona occludins-1 and junctional adhesion molecule-1 [18]. Taken together these studies suggest that activation of TLRs, such as TLR3 can result in a perturbation of the local environment, specifically dysregulation of the airway epithelium thereby supporting an environment primed for an exacerbation. We are currently focusing efforts in our laboratory toward identifying the composition of the mononuclear cell populations in this model including the activation state of various cell types including dendritic cells. In a review by Fe *et. al. *it is summarized that TLR3 can induce a variety of cytokines in human dendritic cells including IFNβ, and CXCL10 [19].

*In vivo *TLR3 agonism by synthetic dsRNA also resulted in a profound up regulation of the expression and secretion of multiple pro-inflammatory cytokines, chemokines, and growth factors. *In vitro *studies have demonstrated that activation of TLR3 by dsRNA on different cell types including natural killer cells [20], epithelial cells [3,21,22], and smooth muscle cells [14] results in increased expression and/or secretion of pro-inflammatory cytokines including IL-6, CXCL-8, CCL-2, CCL-5, CXCL-10, GM-CSF, TNFα and IFNγ. A likely source of cytokines following poly(I:C) administration may be the airway epithelium since activation of BEAS-2B cells *in vitro *induced a profile of pro-inflammatory cytokines similar to that observed following *in vivo *poly(I:C) challenge. TLR3 has been identified and functionally characterized in mouse tracheal muscle [23] and in primary human small airway epithelial cells [21,3,22]. Previous *in vitro *studies have also demonstrated the secretion of inflammatory mediators following TLR3 activation of epithelial cells[3,3,21]. The up regulation of pro-inflammatory cytokines and chemokines provides an inflammatory milieu supporting the infiltration of inflammatory cells into the airways and lung interstitium. Accompanying the inflammation-rich pathology was the presence of bronchial epithelial cell hypertrophy. The hypertrophic cells extended into the secondary and tertiary airways. Epithelial cell hypertrophy is normally associated with increased mucus production [23]. However, in the current study, there was no evidence for increased mucus production by PAS staining. Given the distribution of goblet cells in normal mouse airways, which is restricted to the main bronchi and primary bronchioles, the data suggest that the hypertrophic epithelial cells are not mucus-producing goblet cells.

Along with the demonstration that poly(I:C), acting as a TLR3 ligand, results in an inflammatory response *in vivo*, the study presents a novel finding that stimulation of TLR3 results in a measurable impairment of lung function both without provocation and characterized by increased AHR to methacholine. Similar changes in baseline lung function have also been described in mice exposed to Respiratory Syncytial virus (RSV) [24]. Recent studies have demonstrated that pre-exposure of mouse tracheas to poly(I:C) in vitro increases the expression of bradykinin B1 and B2 receptors on the smooth muscle and confers AHR to bradykinin [25]. Notably, inhibition of the bradykinin B1 receptor confers protection from acetylcholine-induced AHR following allergen sensitization and challenge [26]. In contrast, AHR to histamine following *parainfluenza-3 *infection in guinea pigs was inhibited by a bradykinin B2 receptor antagonist [27]. Taken together these data suggest a role for bradykinin in TLR3-induced airway dysfunction. In the current study some, but not all, functional responses were protected in TLR3 KO mice following multiple administrations of poly(I:C). Specifically, they were protected from baseline lung function changes in response to poly(I:C), however protection from AHR in response to provocation with methacholine did not result in significant protection. Further, the pro-inflammatory mediators produced following poly(I:C) administration were not modulated in TLR3 KO mice. Unpublished data from our laboratory has shown that TLR3 KO mice were significantly protected from a single administration of poly(I:C) with respect to pro-inflammatory mediators in the bronchoalveolar lavage (data not shown), indicating that mediators released in response to acute activation with poly(I:C) may be more TLR3 dependent. This data suggests that another receptor for poly(I:C) may be available. Indeed, since a percentage of TLR3 KO mice succumb to poly(I:C)-induced shock, it suggests that poly(I:C) may still signal in the absence of TLR3 [1]. Indeed, dsRNA can also signal through dsRNA-dependent protein kinase (PKR) [28], RIGI [29] and MDA-5 [30]. The potential redundancy in the dsRNA downstream pathways may be an explanation for the incomplete protection observed in TLR3 KO mice.

Understanding the different signaling pathways involved in recognition of dsRNA by the host has been a major area of focus by many researchers. Le Goffic *et al. *demonstrated that sensing of *influenza A virus *by TLR3 and RIG-I regulates a pro-inflammatory response. In contrast, RIG-I but not MDA-5 also mediates type I IFN-dependent antiviral signaling response[31]. Use of non-poly(I:C) TLR3 ligands is necessary to further define the impact of TLR3-specific signaling on pulmonary pathophysiology. Interestingly, TLR3 KO mice demonstrate protection from *influenza A *virus-induced lung function impairment accompanied by reduced inflammation and improved survival [32].

These data taken along with the inflammatory consequences of TLR3 activation suggest that sustained TLR3 activation may also contribute to severe exacerbations of chronic pulmonary diseases. In summary, the data presented in this study suggest that sustained TLR3 activation may play an important role in respiratory disease pathogenesis. A better understanding of the effects of TLR3 activation will provide additional insight into the mechanisms underlying virus-induced exacerbations associated with respiratory diseases. Additionally, these studies provide an opportunity to identify suitable targets for therapeutic intervention for respiratory disease exacerbations.

## Competing interests

NCS, JS, HAR, KAS, RJL, DDE, PJB, LAM, PA M, RAB, LRS, DEG, RTS, MLM, and AMD are current or former employees of Centocor Research & Development, Inc. RAF and LA declare that they have no competing interests.

## Authors' contributions

NCS conceived of the study and participated in its design and coordination as well as all analysis. JS, LAM, LRS, DEG, RTS, MLM, and AMD participated in the design and coordination of the studies. HAR, and KAS executed the in-life portion of the studies. RJL carried out the BEAS2B studies. DDE and PJB carried out the histopath analysis of the lungs. PAM carried out the statistical analysis of all data sets. RAB carried out the analysis of cellular infiltrates in the lung. RAF and LA made the TLR3 KO mice and gave input on the design of the studies and the manuscript. All authors read and approved the final manuscript.

## References

[B1] Alexopoulou L, Holt AC, Medzhitov R, Flavell RA (2001). Recognition of double-stranded rna and activation of nf-kappab by toll-like receptor 3. Nature.

[B2] Kariko K, Ni H, Capodici J, Lamphier M, Weissman D (2004). Mrna is an endogenous ligand for toll-like receptor 3. J Biol Chem.

[B3] Sha Q, Truong-Tran AQ, Plitt JR, Beck LA, Schleimer RP (2004). Activation of airway epithelial cells by toll-like receptor agonists. Am J Respir Cell Mol Biol.

[B4] Tabeta K, Georgel P, Janssen E, Du X, Hoebe K, Crozat K, Mudd S, Shamel L, Sovath S, Goode J (2004). Toll-like receptors 9 and 3 as essential components of innate immune defense against mouse cytomegalovirus infection. Proc Natl Acad Sci USA.

[B5] Takeda K, Akira S (2004). Microbial recognition by toll-like receptors. J Dermatol Sci.

[B6] Johnston SL (1995). Natural and experimental rhinovirus infections of the lower respiratory tract. Am J Respir Crit Care Med.

[B7] Gern JE, French DA, Grindle KA, Brockman-Schneider RA, Konno S, Busse WW (2003). Double-stranded rna induces the synthesis of specific chemokines by bronchial epithelial cells. Am J Respir Cell Mol Biol.

[B8] Panina-Bordignon P, D'Ambrosio D (2003). Chemokines and their receptors in asthma and chronic obstructive pulmonary disease. Curr Opin Pulm Med.

[B9] Guillot L, Le Goffic R, Bloch S, Escriou N, Akira S, Chignard M, Si-Tahar M (2005). Involvement of toll-like receptor 3 in the immune response of lung epithelial cells to double-stranded rna and influenza a virus. J Biol Chem.

[B10] Hewson CA, Jardine A, Edwards MR, Laza-Stanca V, Johnston SL (2005). Toll-like receptor 3 is induced by and mediates antiviral activity against rhinovirus infection of human bronchial epithelial cells. J Virol.

[B11] Matsukura S, Kokubu F, Kurokawa M, Kawaguchi M, Ieki K, Kuga H, Odaka M, Suzuki S, Watanabe S, Takeuchi H (2006). Synthetic double-stranded rna induces multiple genes related to inflammation through toll-like receptor 3 depending on nf-kappab and/or irf-3 in airway epithelial cells. Clin Exp Allergy.

[B12] Yamamoto M, Sato S, Hemmi H, Hoshino K, Kaisho T, Sanjo H, Takeuchi O, Sugiyama M, Okabe M, Takeda K (2003). Role of adaptor trif in the myd88-independent toll-like receptor signaling pathway. Science.

[B13] Yamamoto M, Sato S, Mori K, Hoshino K, Takeuchi O, Takeda K, Akira S (2002). Cutting edge: A novel toll/il-1 receptor domain-containing adapter that preferentially activates the ifn-beta promoter in the toll-like receptor signaling. J Immunol.

[B14] Sukkar MB, Xie S, Khorasani NM, Kon OM, Stanbridge R, Issa R, Chung KF (2006). Toll-like receptor 2, 3, and 4 expression and function in human airway smooth muscle. J Allergy Clin Immunol.

[B15] Zarember KA, Godowski PJ (2002). Tissue expression of human toll-like receptors and differential regulation of toll-like receptor mrnas in leukocytes in response to microbes, their products, and cytokines. J Immunol.

[B16] Hayashi F, Means TK, Luster AD (2003). Toll-like receptors stimulate human neutrophil function. Blood.

[B17] Sajjan US, Jia Y, Newcomb DC, Bentley JK, Lukacs NW, LiPuma JJ, Hershenson MB (2006). H. Influenzae potentiates airway epithelial cell responses to rhinovirus by increasing icam-1 and tlr3 expression. Faseb J.

[B18] Sajjan US, Wang Q, Jia Y, LiPuma J, Hershenson MB (2007). Rhinovirus compromises tight junctions in differentiated airway epithelial cells and predisposes mice to secondary bacterial infections. Am J Respir Crit Care Med.

[B19] Re F, Strominger JL (2004). Heterogeneity of tlr-induced responses in dendritic cells: From innate to adaptive immunity. Immunobiology.

[B20] Schmidt KN, Leung B, Kwong M, Zarember KA, Satyal S, Navas TA, Wang F, Godowski PJ (2004). Apc-independent activation of nk cells by the toll-like receptor 3 agonist double-stranded rna. J Immunol.

[B21] Ritter M, Mennerich D, Weith A, Seither P (2005). Characterization of toll-like receptors in primary lung epithelial cells: Strong impact of the tlr3 ligand poly(i:C) on the regulation of toll-like receptors, adaptor proteins and inflammatory response. J Inflamm (Lond).

[B22] Ieki K, Matsukura S, Kokubu F, Kimura T, Kuga H, Kawaguchi M, Odaka M, Suzuki S, Watanabe S, Takeuchi H (2004). Double-stranded rna activates rantes gene transcription through co-operation of nuclear factor-kappab and interferon regulatory factors in human airway epithelial cells. Clin Exp Allergy.

[B23] Rogers DF (2004). Airway mucus hypersecretion in asthma: An undervalued pathology?. Curr Opin Pharmacol.

[B24] Schwarze J, Schauer U (2004). Enhanced virulence, airway inflammation and impaired lung function induced by respiratory syncytial virus deficient in secreted g protein. Thorax.

[B25] Bachar O, Adner M, Uddman R, Cardell LO (2004). Toll-like receptor stimulation induces airway hyper-responsiveness to bradykinin, an effect mediated by jnk and nf-kappa b signaling pathways. Eur J Immunol.

[B26] Huang TJ, Haddad EB, Fox AJ, Salmon M, Jones C, Burgess G, Chung KF (1999). Contribution of bradykinin b(1) and b(2) receptors in allergen-induced bronchial hyperresponsiveness. Am J Respir Crit Care Med.

[B27] Folkerts G, Vlieger JW, de Vries A, Faas S, Linde H van Der, Engels F, de Jong JC, Verheyen FA, Van Heuven-Nolsen D, Nijkamp FP (2000). Virus- and bradykinin-induced airway hyperresponsiveness in guinea pigs. Am J Respir Crit Care Med.

[B28] Clemens MJ, Elia A (1997). The double-stranded rna-dependent protein kinase pkr: Structure and function. J Interferon Cytokine Res.

[B29] Yoneyama M, Kikuchi M, Natsukawa T, Shinobu N, Imaizumi T, Miyagishi M, Taira K, Akira S, Fujita T (2004). The rna helicase rig-i has an essential function in double-stranded rna-induced innate antiviral responses. Nat Immunol.

[B30] Gitlin L, Barchet W, Gilfillan S, Cella M, Beutler B, Flavell RA, Diamond MS, Colonna M (2006). Essential role of mda-5 in type i ifn responses to polyriboinosinic:Polyribocytidylic acid and encephalomyocarditis picornavirus. Proc Natl Acad Sci USA.

[B31] Le Goffic R, Pothlichet J, Vitour D, Fujita T, Meurs E, Chignard M, Si-Tahar M (2007). Cutting edge: Influenza a virus activates tlr3-dependent inflammatory and rig-i-dependent antiviral responses in human lung epithelial cells. J Immunol.

[B32] Le Goffic R, Balloy V, Lagranderie M, Alexopoulou L, Escriou N, Flavell R, Chignard M, Si-Tahar M (2006). Detrimental contribution of the toll-like receptor (tlr)3 to influenza a virus-induced acute pneumonia. PLoS Pathog.

